# DNA ligase IV mutations confer shorter lifespan and increased sensitivity to nutrient stress in *Drosophila melanogaster*

**DOI:** 10.1007/s13353-021-00637-0

**Published:** 2021-11-24

**Authors:** Rashmi Joshi, Surya Jyoti Banerjee, Jennifer Curtiss, Amanda K. Ashley

**Affiliations:** 1 Department of Chemistry and Biochemistry, New Mexico State University, NM Las Cruces, USA; 2 Department of Biology, New Mexico State University, NM Las Cruces, USA

**Keywords:** *Drosophila melanogaster*, DNA ligase IV, Lifespan, Starvation

## Abstract

The nonhomologous end-joining pathway is a primary DNA double-strand break repair pathway in eukaryotes. DNA ligase IV (Lig4) catalyzes the final step of DNA end ligation in this pathway. Partial loss of *Lig4* in mammals causes Lig4 syndrome, while complete loss is embryonically lethal. *DNA ligase 4* (*DNAlig4*) null *Drosophila melanogaster* is viable, but sensitive to ionizing radiation during early development. We proposed to explore if *DNAlig4* loss induced other long-term sensitivities and defects in *D. melanogaster.* We demonstrated that *DNAlig4* mutant strains had decreased lifespan and lower resistance to nutrient deprivation, indicating Lig4 is required for maintaining health and longevity in *D. melanogaster*.

## Introduction

DNA damage is a major hallmark of cancer (Hanahan and Weinberg (2011)), and aberrations in pathways maintaining genomic fidelity are associated with multiple cancers (Brown 2017). In mammals, DNA double-strand breaks (DSB) are primarily repaired by homologous recombination (HR) and classical nonhomologous end-joining (cNHEJ); if cNHEJ is compromised, cells may use alternative NHEJ (altNHEJ). Double-strand breaks are recognized by the Ku70-Ku80 dimer, which recruits DNA-PKcs, Artemis, and DNA ligase IV (Lig4) with XRCC4 (Lieber 2010). Lig4 is an ATP-dependent ligase that catalyzes the phosphodiester bond formation in cNHEJ-mediated DSB repair (Lieber 2010), and, in contrast to DNA ligases I and III which facilitate homeostatic DNA metabolism, the activity of Lig4 is restricted to cNHEJ (Lieber 2010).

*Drosophila. melanogaster* has been extensively used as a model to study DNA repair. The DSB repair in *D. melanogaster*, like in mammals, is via the two primary pathways: the template-dependent homologous recombination and template-independent end-joining (EJ) pathway as well as the alternative end-joining (altEJ) pathway (Gorski 2003; Mota 2019). These pathways and corresponding components in *D. melanogaster* are similar to the mammalian system, yet there are some key differences. In the *D. melanogaster* EJ pathway, DSB are recognized by orthologs of the Ku 70/80 heterodimer: Irbp and Ku80. The *D. melanogaster* ligation complex is composed of *D. melanogaster* DNAlig4 and orthologs of XRCC4 (CG3448) and XLF (CG12728 and CG32756). One striking deviation of the *D. melanogaster* EJ is the absence of the key protein, DNA-PKcs, a critical component of the mammalian NHEJ pathway (Mota 2019). *D. melanogaster* EJ pathway also lacks polymerases µ and λ and the nuclease Artemis (recently reviewed (Sekelsky 2017)).

*Lig4* hypomorphic mutations in humans cause the Lig4 syndrome (recently reviewed (Altmann and Gennery 2016)), and *Lig4*^*−/−*^ mice are inviable, as mutations cause p53-mediated neuronal apoptosis resulting in embryonic lethality (Frank 2000). In contrast, *D. melanogaster* males and females lacking *DNAlig4* function are viable and fertile; however, these mutants are hypersensitive to ionizing radiation (IR)–induced DNA damage during early development (Gorski 2003; McVey et al. 2004). An accumulation of DNA damage is commonly observed as a result of aging, and metabolic alterations resulting from nutrient deprivation can induce oxidative stress which in turn induces DNA damage (Filomeni et al. 2015). We wanted to investigate whether mutations in *DNAlig4* modulates *D. melanogaster* longevity and response to nutrient deprivation.

## Results and discussion

We obtained three strains of *D. melanogaster* from the Bloomington Drosophila Stock Center: *w*^*1118*^ (wild-type *DNAlig4*) and two *DNAlig4* mutants: *DNAlig4*^*5*^and *DNAlig4*^*57* (^Gorski 2003). The *D. melanogaster* stocks were maintained at 25 °C on normal yeast-cornmeal food (ingredients: 337.5 g yeast, 195 g soy flour, 1425 g cornmeal, 95 g *Drosophila* food-grade Agar type II, 900 g malt extract, 1.5 g molasses, 100 mL propionic acid, 250 mL 10% Tegosept, and 25 L tap water) unless mentioned otherwise. Flies used for all the experiments were obtained from fertilized eggs laid by homozygous mothers. No differences were observed in fertility or differential distribution of offspring sexes (data not shown).

**Fig. 1:**
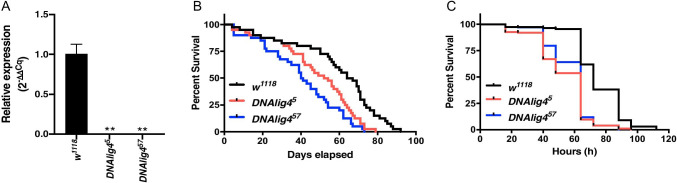
*DNAlig4* mutants have a reduced lifespan and are more sensitive to starvation compared to wild-type *D. melanogaster*. **A** Transcript levels of *DNAlig4* were analyzed by qPCR using primers spanning the first two exons which were deleted in the mutant strains. RNA was harvested from two groups of 10 flies each of mixed sexes. The RNA expression for each of the *DNAlig4* mutants was each compared to the wild-type (*w*^*1118*^) flies. **p < 0.01, *n* = 2. **B** Lifespan was assessed in wild-type (*w*^*1118*^) and mutant *DNAlig4* flies. Median lifespan (expressed as days post emergence) is listed in Table 1 (*p* = 0.001 for *w*^*1118*^ compared to *DNAlig4*^*5*^, *p* < 0.0001 for *w*^*1118*^ compared to *DNAlig4*^*57*^, *n* = 40 flies per strain). **C** Survival under starvation conditions was assessed as mentioned in the Results and discussion section. Median survival (expressed as hours into starvation) is listed in Table 1 (*p* < 0.001 for *w*^*1118*^ compared to *DNAlig4*^*5*^, *p* < 0.001 for *w*^*1118*^ compared to and *DNAlig4*^*57*^, *n* = 100 flies per strain)

We confirmed that *DNAlig4* mutant strains do not produce *DNAlig4* transcripts using qPCR. We designed *DNAlig4* primers that recognize the first two exons (completely deleted in the mutant strains). Ten flies of each gender per strain were used for RNA isolation. After homogenization, RNA isolation was accomplished using the trizol-chloroform-ethanol method (Sigma-Aldrich, St. Louis, MO). We used standard conditions for qPCR (detailed here (Joshi et al. 2019)), except that the annealing temperature was adjusted to 60 °C. Primer sets were designed to recognize full-length *DNAlig4* transcript. *DNAlig4* mRNA expression was assessed by normalizing to *Rp49* (*Ribosomal protein L32* (*RpL32)*). Primer sequences used are as follows: *DNAlig4* (full-length *DNAlig4* mRNA): 5′ GGACACTGGTCGGGATACCT 3′, 5′CTGATGGCATCGCTGGAGTC 3′; and *Rp49*: 5′ CCAAGCACTGCATCCGCCACC 3′, 5′ GCGGGTGCGCTTGTTCGATCC 3′. *DNAlig4* Cq values were normalized to Cq values of *Rp49* via the 2^−ΔΔCq^ method (Livak and Schmittgen 2001) and analyzed via one-way ANOVA with an ad hoc Dunnett’s multiple comparisons test using the statistical software GraphPad Prism. We observed that Cq values for the housekeeping gene for the three strains fell within one cycle of each other. The relative expression of *DNAlig4* in *w*^*1118*^ was 1.004, while in *DNAlig4*^*5*^ and *DNAlig4*^*57*^, mutant strain expression was 0.0008 or 0.0009, respectively (Fig. 1A), validating the *DNAlig4* mutations result in no *Lig4* transcripts.

Next, we evaluated the impact of *DNAlig4* loss on lifespan. Adult male and female flies were mated, and embryos were harvested. Per genotype, 100 embryos were collected using forceps and a dissecting microscope and placed into vials containing approximately 10 mL of standard fly food and stored at 25 °C for 10 days until eclosion. Freshly emerged male and female virgin flies were collected and stored in groups of 10, into vials containing approximately 10 mL of standard fly food. Flies were routinely transferred to fresh vials using CO_2_ to anesthetize during transfer. The number of dead flies was recorded daily until all flies were dead. A total of 40 flies per strain were assessed. Kaplan–Meier survival curves were created for survivorship using GraphPad Prism, and statistical significance was calculated using the Mantel-Cox log rank test. We observed that *w*^*1118*^ flies survived the longest with a median survival of 65.5 days (Table 1). Both the *DNAlig4* mutants had significantly shorter lifespans: median survival for mutant flies was 53 days and 40.5 days for the *DNAlig4*^*5*^ and *DNAlig4*^*57*^ strains, respectively, and both *DNALig4* mutant fly strains were statistically similar to one another (Fig. 1B and Table 1). Our results are in agreement with a previous study where a different *DNAlig4* mutant, the *DNAlig4*^*169a*^, was shown to have reduced lifespan compared to the wild-type strain (Garcia 2011).

**Table 1:** Median lifespan of *DNAlig4* wild-type and mutant flies and median survival under conditions of nutrient deprivation. Survival curves were obtained using GraphPad Prism; *p* values were calculated using Mantel-Cox test

	w^*1118*^	*DNAlig4*^*5*^	*p* value versus w^*1118*^	*DNAlig4*^*57*^	*p* value versus w^*1118*^
Median lifespan	65.5 d	53 d	*p* = 0.001	40.5 d	*p* < 0.0001
Median survival	72 h	64 h	*p* < 0.001	64 h	*p* < 0.001

We investigated the effect of nutrient deprivation on the survival of *DNAlig4* mutants. Briefly, age-matched flies were mated as above, and freshly emerged male and female virgin flies were collected and cultured in groups of 10 into vials containing approximately 10 mL of standard fly food for 48 h. Flies were then transferred to vials with 1% agar with water. Dead flies were scored twice a day (in a cycle of 16 h and 8 h intervals) until all the flies died; 100 flies per strain were assessed. Survival curves were created for survivorship and statistically analyzed as above. *w*^*1118*^ flies were significantly more resistant to nutrient deprivation than either of the *DNAlig4* mutant strains, which, again, were statistically similar to one another (Fig. 1C and Table 1).

The reduced lifespan we observed in *DNAlig4* mutants is likely a function of accumulation of unresolved DNA damage. We attribute the diminished capacity to withstand nutrient stress to induction of oxidative DNA damage that is resolved with less efficiency in *DNAlig4* mutant strains. Overall, we can conclude that while loss of *DNAlig4* does not hinder viability of progeny from homozygous mutant parents or manifest any obvious phenotypic defects in *D. melanogaster*, it does negatively impact the lifespan of adult flies and sensitizes them to nutrient deprivation. We conclude that DNA Lig4 is required for maintaining health and longevity in *D. melanogaster*; the role of DNA Lig4 in supporting health and lifespan of other organisms is currently unknown.
